# New efficacy of LTRAs (montelukast sodium): it possibly prevents food-induced abdominal symptoms during oral immunotherapy

**DOI:** 10.1186/1710-1492-10-3

**Published:** 2014-01-17

**Authors:** Masaya Takahashi, Shoichiro Taniuchi, Kazuhiko Soejima, Kyoko Sudo, Yasuko Hatano, Kazunari Kaneko

**Affiliations:** 1 Department of Pediatrics, Kansai Medical University, 10-15 Fumizono-cho, Moriguchi -shi, Osaka 570-8506, Japan

**Keywords:** Abdominal pain, Food allergy, LTRA (montelukast sodium), Rush oral immunotherapy

## Abstract

**Background:**

The aim of the study was to elucidate whether leukotriene receptor antagonists (LTRAs) can prevent severe allergic reactions, which occur during oral immunotherapy (OIT) in children with food allergies.

**Findings:**

Five children with food allergies [3 allergic to hen’s egg (HE), 1 to wheat, and one to cow’s milk (CM); aged between 7 and 12 years; median, 8.5 years] who were started on LTRAs during OIT were retrospectively selected from among 63 children undergoing OIT. In the rush phase, after the administration of the initial dose which was set in open food challenge test, the subsequent doses were increased by approximately 1.2 times of the previous dose and were administered every 2 hours, 4 times a day. The target doses of hen’s egg, wheat (udon noodle), and cow’s milk in the rush phase were 50 g, 200 g, and 200 ml, respectively. The ingestion of the target dose was continued at home every day for at least a year in the maintained phase.

Four participants experienced intractable abdominal pain during the rush phase; therefore, the loading dose was not increased in these children. However, the administration of LTRAs prevented their symptoms, resulting in the completion of the rush phase. One participant also experienced intractable abdominal pain during the maintenance phase. After receiving LTRAs, the target dose was able to tolerated.

**Conclusion:**

The findings from this retrospective study suggest that the administration of LTRAs is useful for the prevention of adverse allergic reactions such as abdominal pain during OIT.

## Findings

Food allergy is defined as an adverse response to a specific food antigen that is initiated by the immune system. The only current treatment for food allergy is avoidance, while maintaining the hope of outgrowing it over time. Although some trials of oral immunotherapy (OIT) in patients with food allergy have been carried out, the rate of tolerance induction remains uncertain, and food-induced allergic symptoms frequently occur [1].

Eosinophilic gastroenteritis (EG) has been strongly associated with food allergy, and concomitant atopic disease or a family history of allergy is elicited in about 70% of cases [2].

Leukotriene receptor antagonists (LTRAs) alleviate the symptoms of many allergic diseases, including respiratory symptoms such as bronchial asthma [3] and abdominal symptoms such as those occurring during EG [4].

During OIT, patients are sometimes not able to continue the therapy due to severe allergic symptoms, especially intractable abdominal pain, repeated vomiting, and diarrhea. Antihistamines and LTRAs have preventive effects in urticaria and wheezing, respectively; however, an optimal treatment for abdominal symptoms that occur during OIT has not been established.

In the present study, we described the cases of 5 children who successfully continued OIT after the introduction of LTRAs.

A retrospective survey of the medical records of 63 children who underwent OIT between 2010 and 2011 at Kansai Medical University was performed. Children who received LTRAs from the start of OIT were excluded from the study. We identified the medical records of 5 children with food allergies [3 allergic to hen’s egg (HE), 1 to wheat, and one to cow’s milk (CM)] who were started on LTRAs during OIT, these patients were enrolled in the study.

The OIT was performed according to Itoh’s protocol [5]. Before OIT, a double-blind, placebo control food challenge was performed, and all five participants had positive challenges. All five were hospitalized during the rush phase of OIT as per protocol. The target doses of HE, wheat (noodles), and CM in the rush phase were 50 g, 200 g, and 200 ml, respectively. Ingestion of the target dose was continued at home every day to maintain the effect of OIT for at least 6–12 months in the maintenance phase.

All study protocols were approved by the Institutional Ethics Committee of Kansai Medical University.

Data for each of the five participants, who were each started LTRAs during OIT, are summarized in Table 1. All five developed severe abdominal pain and were unable to continue OIT before receiving LTRAs. The clinical course of each participant is summarized below.

**Table 1:** Summary of the participants

**Case**	**1**	**2**	**3**	**4**	**5**
Age (years)	11	8	12	8	7
Sex (male/female)	M	M	M	M	F
Food	Egg	Egg	Egg	Wheat	Cow’s milk
Study phase at the start of LTRAs administration	Rush	Rush	Rush	Rush	M. P.
Frequencies/dose of AS before administration of LTRAs	0.47	0.57	0.29	0.50	0.93
Duration (days) of persistent AS before administration of LTRAs	6	4	5	4	7
Specific IgE level (U_A_/mL) before administration of LTRAs	25.1	12.7	9.61	78.1	0.90
Specific IgE level (U_A_/mL) after administration of LTRAs	19.7	12.0	7.86	52.2	0.73
Treatment period (months) of LTRAs	5	6	2	5	5

LTRAs: leukotriene receptor antagonisits.

M.P.: maintenance phase.

AS: abdominal symptoms.

### Case 1: HE allergic

OIT was initiated at 0.001 g of cooked egg. Repeated, severe abdominal symptoms frequently are redundant during the rush phase; therefore, the dose of egg could not be increased. It remained 1.5 g at 27 days after initiation of OIT. After administration of LTRAs, severe abdominal symptoms no longer occurred with any frequency, and the target dose was achieved after 22 days.

### Case 2: HE allergic

OIT was initiated at 1.0 g of cooked egg. The dose increased to 8.6 g with no severe adverse reactions for five days. However, for the following three days, OIT could not proceed due to repeated, severe abdominal symptoms. After administration of LTRAs, abdominal symptoms resolved completely, and the target dose could be tolerated six days later.

### Case 3: HE allergic

OIT was initiated at 0.001 g of cooked egg. The dose increased to 2.6 g with no severe adverse allergic reaction for 15 days. However, for the following five days, OIT could not proceed due to repeated, severe abdominal symptoms. Although the patient subsequently experienced mild abdominal symptoms once, the target dose was well-tolerated 5 days after the administration of LTRAs.

### Case 4: wheat allergic

OIT was initiated at 0.6 g of udon noodle. The dose increased to 3.1 g with no severe adverse allergic reaction for four days. However, for the following three days, OIT could not proceed due to repeated, severe abdominal symptoms. Although the patient subsequently experienced mild abdominal symptoms twice, the target dose was well-tolerated 10 days after the administration of LTRAs.

### Case 5: CM allergic

During the rush phase of OIT, the participant experienced no severe side effects and the target dose was ultimately reached for 11 days. However, 49 days after the end of the rush phase, the dose was decreased to 10 ml due to intractable abdominal pain and diarrhea. These symptoms resolved completely after LTRAs administration, and, by gradually increasing the dose by 1 ml each day, the target dose was tolerated for seven months, even though intractable abdominal pain was experienced three times and diarrhea twice during this time.

All participants were able to continue the maintenance dose without severe allergic abdominal symptoms after the introduction of LTRAs. Finally, more than one year after the start of OIT, they were able to maintain the target dose of each food without the need for LTRAs.

In the OIT literature, food-induced allergic symptoms are reportedly seen in 50-60% of cases [6,7]. Longo et al. [8] also reported that 23 of 30 children with CM allergy in their OIT group experienced food-induced allergic abdominal symptoms.

LTRAs are used to treat the abdominal symptoms of EG. Although the occurrence of EG is generally very low, it frequently develops during OIT. However, to the best of our knowledge, the effectiveness of LTRAs for food-induced allergic abdominal symptoms has yet to be reported. In the present study, we focused on the efficacy of LTRAs for severe food-induced allergic abdominal symptoms during OIT, which was often interrupted because of these symptoms. All participants were given LTRAs, and OIT was subsequently tolerated; all achieved the target dose and maintained OIT. This result suggests that LTRA controls food-induced severe allergic abdominal symptoms. The mechanism of LTRA efficacy during OIT may be explained by the inhibition of eosinophilic activation [9] and degranulation of mast cells, both of which are triggered by leukotriene [10].

Four studies have reported the occurrence of eosinophilic esophagitis (EE) during OIT [11-14]. However only one study showed that the causative food directly induces EE during OIT for the children with CM allergy, and the frequency of EE during OIT is 2.72% (3 out of 110 patients) [14]. In the present study, EE was not observed in the 63 patients [24 allergic to hen’s egg (HE), 12 to wheat, and 27 to cow’s milk (CM)] receiving OIT; the patients did not show typical symptoms of EE such as general weakness, low physical activity, sleep disturbance, and slow growth, retrosternal pain, dysphagia, and abdominal pain immediately after the ingestion of causative food. And also 10 out of the 63 patients had already received LTRA because of the treatment for persistent asthma. The difference may be because of the preventive effect of LTRAs in the development of EE or different causative food; thus, LTRAs are effective in the treatment of EE [15].

Although a larger, double-blind, placebo controlled study is needed to confirm the current results, we suggest that LTRA may prove to be therapeutically efficacious for treatment of food-induced severe allergic abdominal symptoms during OIT.

## Competing interests

The authors declare that they have no competing interests.

## Authors’ contributions

MT and ST conceived the study, designed the study and wrote the paper. KS and YH participated in its design and writing. KS helped to edit the manuscript. KK helped to collect data for analysis. All authors read and approved the final manuscript.
